# Bamboo expansion promotes radial growth of surviving trees in a broadleaf forest

**DOI:** 10.3389/fpls.2023.1242364

**Published:** 2023-09-13

**Authors:** Chao Gong, Xiaoxia Zeng, Xianglong Zhu, Wenhui Huang, Zacchaeus G. Compson, Zewen Ren, Huan Ran, Qingni Song, Qingpei Yang, Dongmei Huang, Jun Liu

**Affiliations:** ^1^ Jiangxi Province Key Laboratory for Bamboo Germplasm Resources and Utilization, College of Forestry, Jiangxi Agricultural University, Nanchang, China; ^2^ Department of Scientific Research, Administration of Jiangxi Qiyunshan Nature Reserve, Ganzhou, China; ^3^ Department of Biological Sciences, Advanced Environmental Research Institute, University of North Texas, Denton, TX, United States; ^4^ School of Humanities and Public Administration, Jiangxi Agricultural University, Nanchang, China

**Keywords:** intra-annual increment, radial growth rate and duration, diel growth patterns, biological expansions, bamboo (*Phyllostachys edulis*)

## Abstract

**Introduction:**

Considerable evidence indicates that some trees are more vulnerable than others during bamboo (*Phyllostachys edulis*) expansion, which can affect plant community structure and alter the environment, but there has been insufficient research on the growth status of surviving individuals in colonized forests.

**Methods:**

In this study, we compared the annual growth increment, growth rate, and onset, cessation, and duration of radial growth of *Alniphyllum fortunei*, *Machilus pauhoi*, and *Castanopsis eyrei* in a bamboo-expended broadleaf forest (BEBF) and a bamboo-absent broadleaf forest (BABF) using high-resolution point dendrometers.

**Results:**

We found that the annual radial growth of *A. fortunei*, *M. pauhoi*, and *C. eyrei* was 22.5%, 172.2%, and 59.3% greater in BEBF than in BABF, respectively. The growth rates of *M. pauhoi* and *C. eyrei* in BEBF were significantly higher than in BABF by13.9 μm/d and 19.6 μm/d, whereas *A. fortunei* decreased significantly by 7.9 μm/d from BABF to BEBF. The onset and cessation of broad-leaf tree growth was later, and the growth duration was longer in BEBF compared to BABF. For example, *A. fortunei* and *M. pauhoi* in BEBF had more than one month longer growth duration than in BABF. Additionally, the nighttime growth rates of some surviving broad-leaf trees in BEBF was significantly higher than that in BABF.

**Discussion:**

These results suggest that the surviving trees have plasticity and can adapt to atmospheric changes and competitive relationships after expansion of bamboo in one of two ways: by increasing their growth rates or by modifying onset and cessation of growth to extend the growth duration of trees or avoid the period of intense competition with bamboo, thereby growing better. Our research reveals for the first time how the growth of surviving broad-leaf trees adjusts to bamboo expansion. These results provide insights into how biological expansions impact primary production and have implications for forest management in the Anthropocene.

## Highlights

(1) Bamboo (*Phyllostachys edulis*) expansion facilitates surviving broadleaf tree growth rates, the duration of the growth phase, and—by altering the growth phase—reducing the period of intense competition with trees in the invaded forest.

(2) Surviving trees exhibit plasticity, can adapt to atmospheric changes, and grow faster than trees in forests not impacted by bamboo expansion despite the fact they are in direct competition with bamboo.

## Introduction

Plants invasion, non-native species invaded and established to native ecosystem that disturb the functioning and stability of these ecosystems (e.g. alter species composition and decrease biodiversity) (Mack et al., 2000; Vilà et al., 2011; Pagad, 2016), are an important component of global change worldwide (Pyšek et al., 2020). Bamboo (*Phyllostachys edulis*), a typical native invasive species (Bai et al., 2016), is characterized for its grows quickly and has a robust rhizome system (Lima et al., 2012; Xu et al., 2020) that enables unbridled invasion (also known as expansion) into adjacent forests, especially secondary broadleaf forests (Ying et al., 2016). Like other invasive alien plant species, bamboo can induce impacts on biodiversity and eco-function. For example, bamboo expansion can hinder the growth of broadleaf trees, leading to the death of some trees and affecting species diversity (Ouyang et al., 2016; Song et al., 2017; Xu et al., 2020). Interestingly, bamboo expansion into broadleaf forests does not eliminate all trees, as trees with narrower crowns or deeper roots can often survive (Zhou et al., 2016), such as *Alniphyllum fortunei* and *Castanopsis fargesii* (Liao et al., 2002; Huang, 2008). However, little information exists about the effects of bamboo expansion processes on the growth status of these surviving trees. Are these tree species struggling to survive under the suppression of bamboo (temporary survival) or are they experiencing healthy growth (long-term survival)? Tree growth data can help illuminate answers to these questions.

The growth status of trees is mainly reflected in changes in the diameter at breast height (DBH), total height, and leaf biomass. Among these, measuring changes in DBH (radial growth) is simple and accurate. Given that radial growth is closely related to tree growth traits, such as tree height and leaf biomass (Aiba and Kohyama, 1996; Liu et al., 2017), it is a key indicator for evaluating tree growth vitality (Fonti et al., 2010; Cocozza et al., 2016; Preisler et al., 2021). Stem radial growth is determined by both growth rate and growth duration, which is the time between the onset and cessation of cambium activity (Wang et al., 2018). Growth rate and growth duration are mainly influenced by environmental factors, such as light conditions, soil moisture, and nutrient content (Chase et al., 2016; Zhang et al., 2021). Additionally, competition among species could also affect radial growth of trees (Liu et al., 2018; Tsai et al., 2018). The expansion of bamboo can have both inhibitory and promoting effects on the radial growth of surviving trees, as it alters environmental conditions and competitive relationships.

Several factors make bamboo a particularly strong competitor and effective invader in many forests. First, both growth and reproduction rates are high in new bamboo, which can reach full maturity and maximum body size within 1~2 months after being unearthed (Song et al., 2016b; Zhao et al., 2017). For example, bamboo can produce 2000 ~ 3000 new stems/hm^2^ per year (Li et al., 2000). Additionally, the underground roots of bamboo are vast, and the biomass of fine roots is approximately six times that of broadleaf trees (Liu et al., 2013). Based on these biological characteristics, bamboo has a competitive edge in resource acquisition compared to trees. Because bamboo and broadleaf trees have similar demand for resources (Yang et al., 2017), they may be in strong competition for resources such as water and nutrients, and trees are at a competitive disadvantage, which typically have lower growth rates, later growth onset, and earlier growth cessation (Liu et al., 2018; Tsai et al., 2018). Therefore, we speculate that bamboo expansion may decrease the growth rate of surviving broadleaf trees and shorten their growth duration through competition.

The consequences of bamboo expansion into native forests, however, are not always negative. With the entry of bamboo and the exit of some broadleaf trees during expansion, the local microenvironment will be changed, including increases in light intensity (Liu et al., 2011), soil temperature (Chi et al., 2020), and soil moisture (Shinohara and Otsuki, 2015; Shinohara et al., 2019). Mechanistically, the relatively well-developed root system and narrow crown of bamboo compared to broadleaf trees could cause a reduction of soil erosion in invaded forests (Shinohara et al., 2019). These environmental changes can have a positive effect on the radial growth rate of surviving broadleaf trees. Simultaneously, changes in temperature (Delpierre et al., 2019; Saderi et al., 2019; Sanmiguel-Vallelado et al., 2021) and water availability (Ziaco et al., 2018; Nanayakkara et al., 2019) can also affect the timing of cambium activity. A warm climate with sufficient water can lead to early and delayed cambium activity in spring and autumn, respectively (Tian et al., 2017; Wang et al., 2018; Kaewmano et al., 2022), thereby prolonging the growth duration of trees. Therefore, the expansion of bamboo may also promote the radial growth rate of surviving broadleaf trees and prolong their growth duration *via* changing environmental conditions.

Surviving broadleaf trees may be affected by the combined inhibitory and promotional factors from bamboo expansion; consequently, we require high-precision instruments for long-term monitoring. Point dendrometers enable collection of high spatial- and temporal-resolution (i.e., sub-hour) growth data for trees (Deslauriers et al., 2003a; Stangler et al., 2021). Dendrometer signals are analyzed through mathematical functions (Cocozza et al., 2012; Maatena et al., 2016) or fitting models (Rossi et al., 2003) to extrapolate information about timing, duration, and rate of tree growth. Here, we conducted high-resolution monitoring of the growth of broadleaf trees in both evergreen broadleaf forest (BABF) and bamboo-broadleaf mixed forest (BEBF) within the Qiyunshan National Nature Reserve, Jiangxi Province, China, where bamboo expansion is relatively severe. Our aim was to answer the following scientific questions: (1) Does bamboo expansion suppress or promote the radial growth rate of surviving broadleaf trees? (2) Does the expansion of bamboo lead to changes in the timing and duration of growth in surviving trees? Addressing these scientific questions will provide the first quantitative assessment of the impact of bamboo expansion on tree growth, illuminate how competing ecological processes affecting primary production play out during bamboo expansion, and provide resource managers with insights into how to manage bamboo expansions during the Anthropocene.

## Materials and methods

### Study area

**Figure 1:**
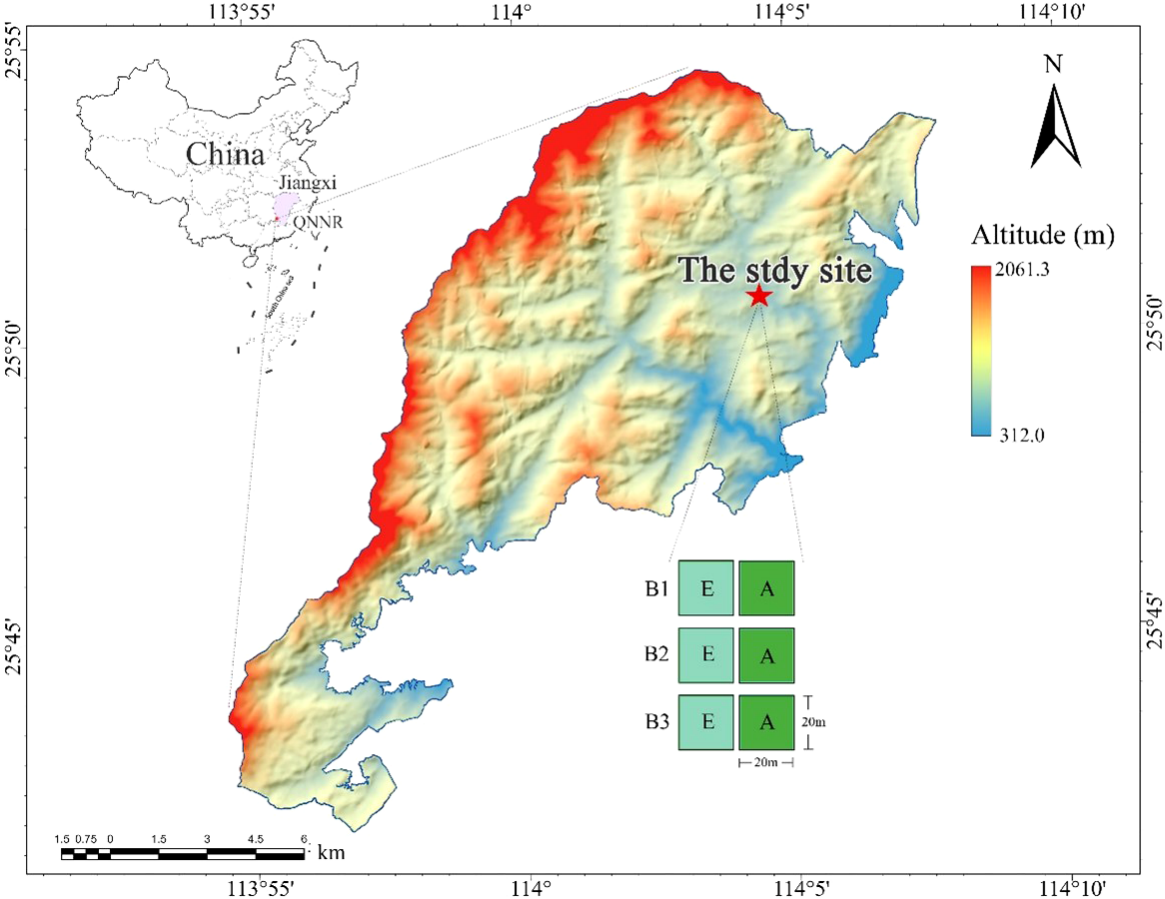
The study location and experimental layout of three blocks (B1; B2; B3). Locations of Jiangxi Province (purple area) and QNNR (red point) are pictured (top left), as well as experimental blocks (red star) in the QNNR area. Bamboo-absent broadleaf forest (A) are depicted by green squares and bamboo-expanded broadleaf forest (E) are depicted by aqua squares.

The study area is located at the Qiyunshan National Nature Reserve (QNNR) in Jiangxi Province, China (113°54′-114°07′E, 25°41′-25°54′N, **Figure 1**), with an elevation of ~2060m a.s.l. QNNR belongs to the humid subtropical monsoon climate zone, which is characterized by a warm climate and abundant rainfall (Yang et al., 2009; Huang et al., 2008). The mean annual precipitation at QNNR ranges from 1522-1660 mm, and the mean annual temperature is between 18.0~18.4 °C (Chen et al., 2019). QNNR’s zonal vegetation is evergreen broadleaf forest, but many bamboo forests remain in some areas. The forest canopy in this area is mainly divided into two layers: the upper canopy is composed of fast-growing deciduous trees and some older evergreen trees (maximum height: 15-20 meters) and the understory mainly consists of adult and young trees of shade-tolerant species.

### Experiment design

**Figure 2:**
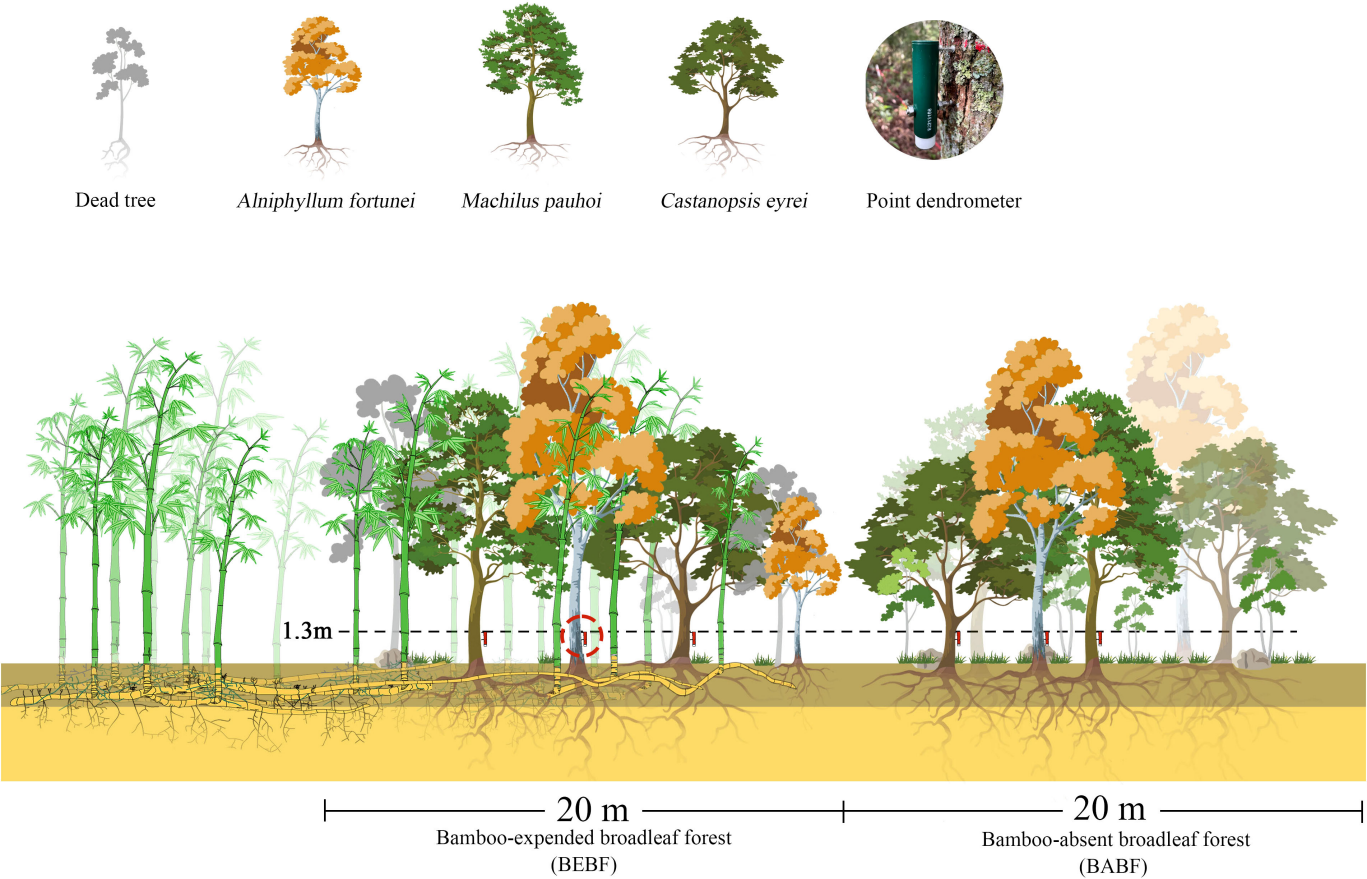
The experimental gradient of bamboo invasion, including the bamboo-expanded broadleaf forest (BEBF) and bamboo-absent broadleaf forest (BABF).

Three experimental blocks, B1, B2, and B3, were established in June 2021 (**Figures 1**, **2**). Each block included a sample plot of bamboo-absent broadleaf forest (BABF, 20 m × 20 m each) and a bamboo-expended broadleaf forest (BEBF, 20 m × 20 m each; *n =* 6 plots total). Sample plots were spaced at distance of >10 m apart. All plots were located on the clay loam northwest slope, with a slope between 16 and 20 degrees. Before the establishment of the nature reserve, the area experienced human-induced disturbances, leading to the formation of secondary broadleaf forests with similar stand densities in each plot. With the establishment of the nature reserve in 1997, the broadleaf forests entered a stage of natural recovery. During the recovery process, the broadleaf forests adjacent to bamboo forests were encroached upon by bamboo expansion, resulting in the formation of bamboo-broadleaf mixed forests.

Average density of trees with DBH greater than 5 cm was ~277 ± 7 and ~135 ± 10 stems per ha for BABF and BEBF in 20 × 20 m plots, respectively. Average density of bamboo was ~0 and ~233 ± 12 stems per ha for BABF and BEBF in 20 × 20 m plots, respectively (**Table 1**). The broad-leaf area included primarily *Alniphyllum fortunei*, *Machilus pauhoi*, *Castanopsis eyrei*, *Castanopsis fargesii*, and *Daphniphyllum oldhamii*, in the bamboo-invaded and bamboo-absent broadleaf forest in our study area. We selected three tree species—*A. fortunei*, *M. pauhoi*, *C. eyrei*—as the focus of our research because they were found in both forest types.

**Table 1:** General information about the bamboo-expended broadleaf forest (BEBF) and bamboo-absent broadleaf forest (BABF).

Stand	Tree			Shrub			Bamboo		
	SD (stem hm^-2^)	DBH (cm)	H (m)	SD (stem hm^-2^)	DBH (cm)	H (m)	SD (stem hm^-2^)	DBH (cm)	H (m)
BABF	277 ± 7	10.00 ± 5.74	7.95 ± 2.86	618 ± 12	2.00 ± 0.91	3.31 ± 1.18	N.D.	N.D.	N.D.
BEBF	135 ± 10	9.52 ± 4.52	7.55 ± 1.59	679 ± 37	2.30 ± 1.19	3.41 ± 1.53	233 ± 12	8.63 ± 1.77	11.10 ± 2.27

SD is the stem density of a stand, DBH is the diameter at breast height, and H is the height of trees. No data (N.D.) were available for bamboo in the BABF forest type because this site was not invaded.

### Collection of climate data

A temperature and humidity recorder (model: TMS5-L30, China, Tomst Company) was installed at the center of each sample plot to collect data of soil temperature at depths of 0 cm (surface) and 6 cm, air temperature at a height of 12 cm, and soil moisture content at a depth of 6 cm.

### Monitoring of tree growth

In each plot, 3 trees with similar diameter size distributions of *A. fortunei*, *M. pauhoi*, and *C. eyrei* were selected for measurement. High resolution point dendrometers were used to monitor stem growth at 15-minute sampling intervals on 18 trees from January 2022 to December 2022, mounted 1.3 m above ground level (diameter at breast height, DBH; **Figure 2**). To reduce the impact of bark expansion and contraction on dendrometer data, we used a knife to carefully remove the outer bark and dead bark tissue without damaging the cambium (Deslauriers et al., 2003b). Basic information of the selected trees and sample plots is demonstrated in **Tables 1** and **2**. Each point dendrometer was individually calibrated in the laboratory before being installed on trees.

**Table 2:** Basic information of measured trees and characterization of growth patterns.

Species	Stand	Diameter at breast height(cm)	Height(m)	Timing of growth initiation	Timing of growth cessation	Growing season duration (days)	Mean seasonal growth rate (μm/d)	Cumulative growth (mm)
*Alniphyllum fortunei*	BABF	13.97 ± 0.97	11.87 ± 1.34	07-May	27-Jul	82	45.47 ± 25.01	4.10 ± 0.84
	BEBF	14.20 ± 0.66	12.67 ± 1.31	10-May	5-Sep	119	37.53 ± 20.71	4.96 ± 0.87
*Machilus pauhoi*	BABF	15.57 ± 0.87	10.47 ± 0.87	06-Apr	27-Jul	113	14.89 ± 7.83	1.86 ± 0.99
	BEBF	14.80 ± 1.05	9.57 ± 0.71	19-Apr	21-Sep	156	28.76 ± 13.28	4.98 ± 1.03
*Castanopsis eyrei*	BABF	13.63 ± 1.29	8.43 ± 1.51	26-Mar	5-Oct	194	25.96 ± 11.76	5.52 ± 1.34
	BEBF	14.28 ± 0.76	8.10 ± 0.92	28-Mar	23-Oct	210	38.06 ± 18.51	8.87 ± 0.80

### Statistical analysis

Data management and analysis were performed using R statistical software (R Core Team, 2019). Raw dendrometer data of each tree, with a 15-minute resolution over the full length of the time series, were quality checked and processed using the R-package TREENETPROC (Knusel et al., 2021). Day and night growth were calculated as the cumulative growth from 6:00 to 18:00 and from 18:00 to 6:00 the next day, respectively, based on hourly-resolved radial stem growth (annual average). We used the sigmoid Gompertz growth model to fit the cumulative daily stem radial growth variation curve, which allowed us to simulate the intra-annual stem radial growth process due to its flexibility and asymmetry (Deslauriers et al., 2003b; Duchesne et al., 2012):


(1) Y=A∗exp[−exp(β−k∗t)]

where Y is the daily stem radial growth maximum, A represents the upper asymptote, *β* is the x-axis placement parameter, *k* is the rate of change parameter, and *t* is time expressed in days.

Based on model results, we calculated the onset and cessation of growth. The growth onset was defined as the day of the year that exceeds the maximum diameter of the previous year, and the growth cessation was considered the day of the year with the largest cumulative growth. To increase comparability between sample trees and reduce the impact of erratic data points during the asymptotic phase of the annual growth curve, the growth onset was defined as the day when the growth exceeded 5% of the yearly growth, and the growth cessation represented the day of the year at which 95% of yearly growth was reached (Mäkinen et al., 2008; Ziaco et al., 2016). The duration of radial increment (i.e., growing season) was the number of days between the onset and cessation of growth. The sum of the radial increment of the growing season was divided by the growth duration to give the mean daily growth rate during the growing season.

We used independent sample t-tests to analyze differences in mean daily growth rate, growth onset and cessation, and radial increment duration of broadleaf trees, as well as air temperature, ground temperature, soil temperature, and soil moisture between BABF and BEBF. The data presented are average values for each species in BABF and BEBF. All statistical analyses were performed using SPSS 24.0, and graphs were created using Origin 2022.

## Results

### Climatic conditions

**Figure 3:**
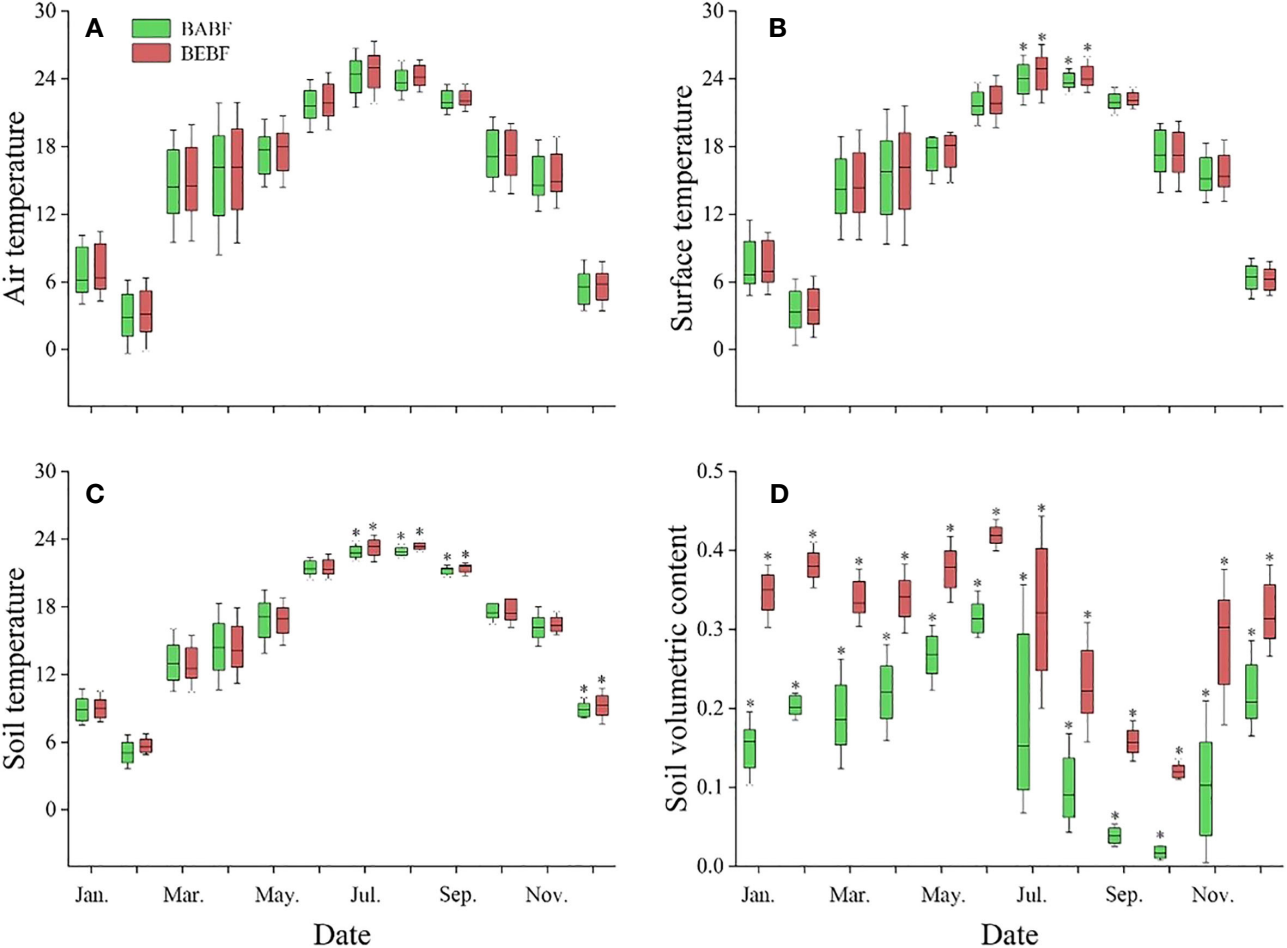
Monthly mean daytime meteorological values for the study period (2022, mean ± S.E.). **(A)** Air temperature, **(B)** surface temperature, **(C)** soil temperature, and **(D)** soil water content (daily means, *n* = 3) for BEBF (red) and BABF (green) forests are depicted. Asterisks designate significant differences between forest types (*α* = 0.05).

Bamboo expansion increased the temperature and soil water content of broadleaf forests, but it had a greater impact on soil water content than on temperature (**Figure 3**). Air temperature (**Figure 3A**), surface temperature (**Figure 3B**), and soil temperature (**Figure 3C**) were generally higher in BEBF compared to BABF, but these differences were not significant in most months; only in July, August, September, and November were they significantly different between sites. Soil water content was significantly higher in BEBF compared to BABF every month; on average, soil water content was 1.3-times higher across all months in BEBF (**Figure 3D**).

### Annual increment

**Figure 4:**
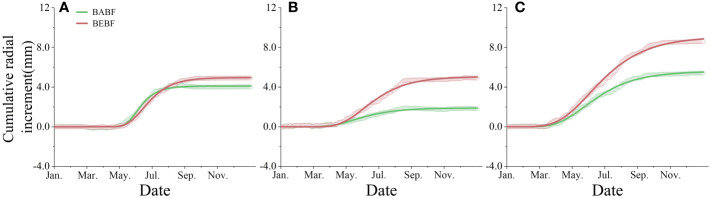
Observed and modeled cumulative radial increment. **(A)** (*A*) *fortunei*, **(B)** *M. pauhoi*, and **(C)** *(C) eyrei* in BEBF (red) and BABF (green) are depicted. Open points and lines indicate the original measured curves and Gompertz function modeled curves (*n* = 3).

Bamboo expansion increased the annual radial increment of surviving broadleaf trees (**Figure 4**). The annual radial increment of *A. fortunei*, *M. pauhoi*, and *C. eyrei* in BABF was 4.0 mm, 1.8 mm, and 5.4 mm, respectively, while in BEBF it was 4.9 mm, 4.9 mm, and 8.6 mm, respectively; bamboo expansion increased their radial growth by 22.5%, 172.2% (*p*<0.05), and 59.3% (*p*<0.05), respectively.

### Growth rates

**Figure 5:**
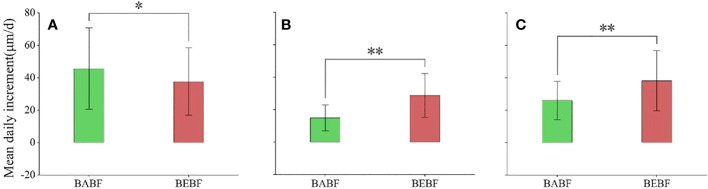
Mean daily growth increment during the growing season. **(A)** (*A*) *fortunei*, **(B)** *M. pauhoi*, and **(C)** *(C) eyrei* in BABF (green) and BEBF (red) are depicted. Error bars denote the 95% confidence interval, and bars labeled with “*” or “**” indicate significant differences at *α* = 0.05, respectively.

The impact of bamboo expansion on the radial growth rate of surviving broadleaf trees varied by species (**Figure 5**; **Table 1**). During the growing season, growth rates of *M. pauhoi*, and *C. eyrei* in the BEBF were about 13.9 μm/d and 19.6 μm/d faster than in the BABF, respectively (all *p<*0.01); however, bamboo expansion decreased growth rates of *A. fortunei* by 7.9 μm/d (*p*< 0.05).

### Onset, cessation, and duration of growth

Bamboo expansion significantly affected the onset, cessation, and duration of growth of surviving broadleaf trees. The growth onset of *A. fortunei*, *M. pauhoi*, and *C. eyrei* occurred about 3 (*p >*0.05), 13 (*p<*0.05) and 2 (*p >*0.05) days later in BEBF compared to BABF forests. Surprisingly, bamboo expansion delayed growth cessation in all trees: *A. fortunei*, *M. pauhoi*, and *C. eyrei* were delayed by 40, 56, and 18 days (all *p<*0.05), respectively (**Table 2**). Additionally, *A. fortunei* and *M. pauhoi* had a significantly longer growing season duration in BEBF compared to BABF (119 compared to 82, 156 compared to 113; both *p<*0.05).

### Diel growth patterns

**Figure 6:**
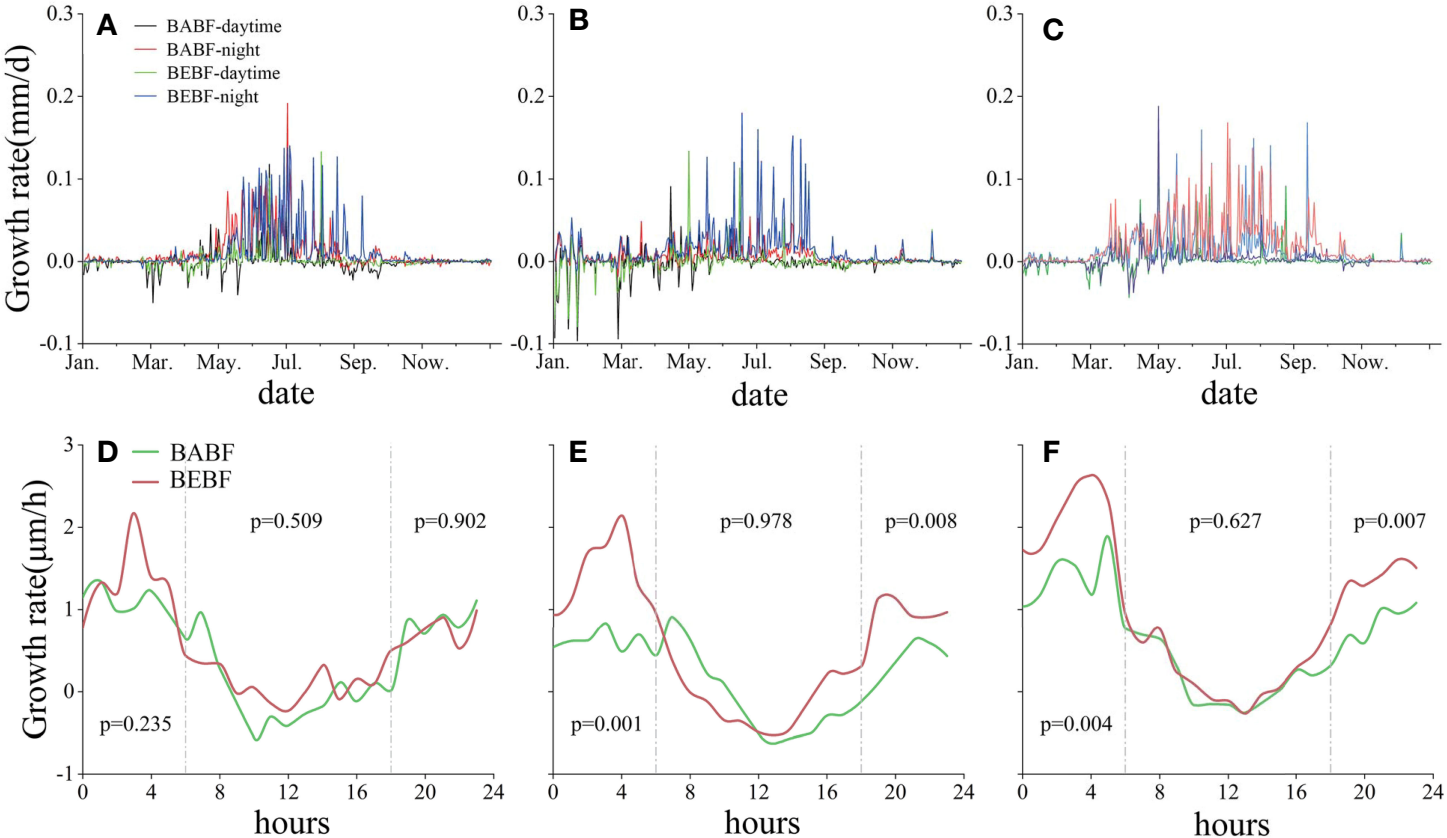
Diel growth rates of three surviving broadleaf tree species. Daytime (BABF, purple; BEBF, green) and nighttime (BABF, red; BEBF, blue) growth rates of *A. fortunei*, *M. pauhoi*, and *C. eyrei* are shown in **(A-C)**. Hourly increments (BABF, green; BEBF, red) of *A. fortunei*, *M. pauhoi*, and *C. eyrei* are shown **(D-F)**, with *p*-values depicting statistical significance of growth increments (*α* = 0.05).

Over a diel cycle, all broadleaf tree species grew mainly at night in BABF and BEBF, with the maximum daily growth rate occurring around 04:00 h and minimum daily growth rate occurring around 11:00 h (**Figure 6**). Although bamboo expansion had no effect on the diurnal growth rhythm of broadleaf trees, it did change the growth per hour of each broadleaf tree species. Specifically, the morning (0:00 h to 6:00 h) and night (16:00 h to 0:00 h) growth rates of *M. pauhoi* and *C. eyrei* increased significantly (**Figures 6E, F**) in BEBF compared to BABF sites.

## Discussion

### Impacts of bamboo expansion on growth rate

Our results showed that bamboo expansion substantially increased radial growth rates of surviving shade-tolerant, evergreen, broadleaf trees (*M. pauhoi* and *C. eyrei*; **Figure 5**), a pattern mostly attributed to changes in environmental factors, such as light and water, that occur when the bamboo-absent broadleaf forests (BABF) are converted to bamboo-expended broadleaf forests (BEBF). It has been reported that light penetration could increase because some trees die during bamboo expansion (Liu et al., 2011), which can promote carbon assimilation (Aussenac, 2000) and lead to increasing radial growth.

Compared to other environmental factors, changes in soil moisture may have a stronger impact on radial growth rates of trees. Our study found that the soil moisture in BEBF was significantly higher than that in BABF (**Figure 3D**). The well-developed root system of bamboo could reduce soil erosion in forests (Shinohara et al., 2019), which could promote water infiltration into the soil and increase soil water content. Furthermore, canopy interception loss in bamboo forests was considerably lower than that of broadleaf forests (Shinohara et al., 2013). Therefore, after the expansion of bamboo, increases in soil moisture content likely replenishes trees at night, promoting expansion and division in cambium cells (Zweifel et al., 2006), conducing to radial growth rates of trees (Primicia et al., 2013). This idea is supported by the nighttime growth rates of trees observed in our study, as the nighttime growth increment of *M. pauhoi* and *C. eyrei* significantly increased from BABF to BEBF (**Figure 6**).

However, expansion of bamboo decreased the growth rate of *A. fortunei* (**Figure 5**). We speculate that this phenomenon was related to water competition. Indeed, *A. fortunei* belongs to shade-intolerant deciduous broadleaf tree species, which have a relatively large specific leaf area (Zeng et al., 2017) and high transpiration rates (Granier et al., 2000) compared to other trees, traits requiring more water than shade-tolerant evergreen tree species (Visser et al., 2018). Thus, the increase in soil moisture caused by bamboo expansion may not have had a significant effect on the growth of *A. fortunei* trees with a high water demand. The root system of *A. fortunei* also experiences stronger competition after the expansion of bamboo because *A. fortunei* trees are shallow-rooted tree species, with roots occurring in soil between 0-15 cm (Xiang et al., 2013). Further, large masses of bamboo roots tend to be distributed at this soil depth; the biomass of bamboo fine roots is approximately six times that of broadleaf trees (Liu et al., 2013), and this dense root network likely outcompetes the root systems of broadleaf trees for water and nutrients. Consequently, in the BEBF *A. fortunei* trees face enormous pressure from water competition, and the stimulated growth brought about by changes in favorable conditions of light, temperature, and water availability is likely insufficient to compensate for the decreased growth caused by competition-driven water shortage.

### Consequences of delayed growth onset and cessation

So why does the growth rate of *A. fortunei* decrease and still survive in BEBF? This is probably related to the regulation of its growth timing. Temperature and water were the main factors determining the onset of tree growth. We found that after bamboo expansion, the onset time of surviving tree growth was delayed (**Table 2**) despite sufficient water and suitable soil and air temperature in the stand (**Figure 3**), which may be influenced by competition from bamboo. We observed that surviving trees faced more competitive pressure in BEBF compared to BABF (**Table S1**), and they may suffer the greatest competitive pressure during spring. Due to the high growth rate of bamboo, it can complete its growth in height and diameter within 1~2 months after shoots emerge from the soil in the spring (Song et al., 2016b; Zhao et al., 2017). During this period, mature bamboo needs to absorb a large amount of water and nutrients *via* underground rhizomes that contribute to new bamboo growth (Li et al., 2000), so trees face greater competitive pressure. When trees are in a highly competitive forests, their growth begins later (Grotta et al., 2005; Liu et al., 2018), likely because intense competition weakens the sensitivity of tree growth to climatic environmental conditions (Wang et al., 2016; Tsai et al., 2018). Our findings reveal that the competitive relationship between bamboo and broadleaf trees may be a major trigger of tree growth initiation under conditions of suitable temperature and sufficient water.

However, the delay in the onset of growth of *A. fortunei* and *C. eyrei* was not significant in our study, likely because the growth of *A. fortunei* started relatively late, in middle of May, during which time new bamboo recruitment was mostly finished (**Table 2**). *C. eyrei*, on the other hand, is a late-stage successional species and has strong competitive ability due to its lower demand for resources and higher utilization efficiency, such as light and water (Kitajima, 1994; Llambí et al., 2003). Additionally, the intensity of intraspecific competition is often higher than that of interspecific competition (Jin et al., 2004); this could explain why *C. eyrei* had no obvious response to bamboo competition in our study. Collectively, these results indicate that the growth initiation time of late-growing or more competitive trees with high resource use is not sensitive to the response of bamboo expansion.

Interestingly, the cessation of growth of all surviving broadleaf tree species was delayed after bamboo expansion, which may be related to changes in temperature and water. Many studies have shown that air temperature and soil temperature are important factors controlling the cessation of cambium activity (Rossi et al., 2016; Nanayakkara et al., 2019). With an increase in temperature, the activity cessation time of cambium cells can be delayed (Gao et al., 2019). We found that soil temperature significantly increased during autumn after bamboo expansion (**Figure 3C**). Generally, soil temperature is influenced by leaf litter layer (e.g., Xu et al., 2013; Fekete et al., 2016), and with a decrease in litter depth, the solar radiation received by the soil increases, leading to an increase in soil temperature. The production and thickness of litter in broadleaf forests can decrease after the expansion of bamboo, especially in summer and autumn (Song et al., 2016a). Thus, after the expansion of bamboo, soil temperature likely increases, resulting in a delay in phenology of cambium activity in the autumn.

Water availability also plays an important role in the cessation of tree radial growth (He et al., 2016). As water availability decreases, drought stress caused by water scarcity can lead to early cessation of radial growth of trees by limiting the cell division rate and reducing photosynthesis (Vieira et al., 2020), as has been shown for several tree species (Zhang et al., 2018; Guillermo et al., 2020; Zhang et al., 2021). We found that the soil moisture content significantly increased, especially in autumn, from BABF to BEBF: the soil moisture content increased from 9.7%, 4.2%, and 2.0% to 22.9%, 16.0%, and 12.0%, respectively (**Figure 2**) in August, September, and October, months when there was almost no rainfall in the study area. Therefore, the increase in soil water availability caused by bamboo expansion can delay the growth cessation of trees. We believe that under arid environmental conditions, the increase in soil moisture caused by bamboo expansion will have a greater positive impact on tree growth timing than the negative impact from competition with bamboo, but this hypothesis requires further study.

Because of the impacts discussed above, it is likely that after bamboo expansion surviving broadleaf trees can coexist stably for a considerable period of time. However, we note that the current coexistence of bamboos and broadleaf trees may not be stable, and their relative frequencies may change in future. When the density of bamboo reaches a certain threshold, this stable state of coexistence may be disrupted; in other words, after exceeding a certain equilibrium density, the positive effects of bamboo on environmental conditions are likely insufficient to compensate for the negative impacts of competition. For example, although bamboo expansion can increase soil moisture content (Shinohara and Otsuki, 2015; Shinohara et al., 2019), some research indicates that bamboo’s transpiration rate is generally higher than that of broadleaf trees (Shi et al., 2007; Shao et al., 2015). Therefore, when bamboo expands past a certain threshold density, its water consumption from the soil may exceed the increase in soil moisture caused by its presence at lower densities. As a result, with further expansion of bamboo, the number of surviving broadleaf tree species may gradually decrease. However, broadleaf trees are unlikely to disappear completely, as some broadleaf tree species in later successional stages have high efficiency in utilizing resources, such as water and light (Kitajima, 1994; Llambí et al., 2003). Further, the intensity of intraspecific competition is often higher than that of interspecific competition (Jin et al., 2004), suggesting that their growth and survival may not have obvious responses to bamboo competition. Additionally, some broadleaf tree species with deep root systems can absorb soil moisture and nutrients from deeper layers, avoiding direct competition with bamboo. Overall, the changes in the relative frequencies of bamboos and broadleaf trees and the underlying mechanisms need further investigation.

After the expansion of bamboo, the diurnal growth rhythm of surviving broadleaf tree species remained unchanged in our study, with growth mainly occurring in the morning and night (**Figure 6**), which is similar to results of other studies (Preisler et al., 2021; Zweifel et al., 2021). On a daily time scale, radial changes in tree growth are closely related to air temperature and stem water conditions (Drew and Downes, 2009). During the daytime, transpiration of trees increases with rising temperatures; when water loss by transpiration exceeds the water absorbed by the roots, the water stored in the trunk is used for transpiration, resulting in shrinkage of the trunk. Water stored in the trunk can support 8~20% of the daily transpiration rate of trees (Cermak et al., 2007). In the early evening, transpiration of trees weakens when the temperature drops, and the water absorbed by roots is higher than that consumed by transpiration, allowing cells to absorb water and expand, leading to stem expansion (Duchesne and Houle, 2011). Findings from our study indicated that the influence of bamboo expansion on environmental conditions and competitive relationships with trees was not sufficient to change the diurnal growth rhythm of surviving broadleaf trees.

## Conclusion

Our study demonstrated that bamboo expansion had a greater promoting effect than inhibiting effect on the radial growth of surviving broadleaf trees, resulting in an increased radial growth increment. For shade-intolerant, deciduous, broad-leaf tree species, bamboo expansion regulated the seasonal growth timing, delaying the cessation of growth and prolonging the growth duration. For shade-tolerant, evergreen species, bamboo expansion not only extended growth duration, but it also facilitated trees in avoiding periods of intense competition with bamboo by changing the onset and cessation time of growth, thereby increasing daily growth rates. Therefore, we believe that the surviving broadleaf trees after bamboo expansion can exist for a long time, and evergreen tree species may exist for an even longer time. Our study highlights the adaptive strategies of surviving broadleaf trees to bamboo expansion and provides insights into how bamboo expansions should be managed during the Anthropocene.

## Data availability statement

The original contributions presented in the study are included in the article/**Supplementary Material**. Further inquiries can be directed to the corresponding author.

## Author contributions

CG and XXZ participated to design of the study, organized the database and wrote the manuscript. XLZ, WH, ZR and HR performed the data collection and statistical analysis. ZC contributed to manuscript revision. QS and QY participated to design of the study and provided funding. DH performed the data collection. JL contributed to conception and design of the study and provided funding acquisition. All authors contributed to the article and approved the submitted version.
