# Supplementary material for: Bamboo expansion promotes radial growth of surviving trees in a broadleaf forest
Source: Front Plant Sci. 2023 Sep 13;14:1242364. doi: 10.3389/fpls.2023.1242364 (PMC10525704; doi:10.3389/fpls.2023.1242364)
Supplement: Supplementary file 1 [file Table_1.docx]

Supplementary Materials

The growth and survival status of trees are usually significantly affected by neighboring trees, and the intensity of impact can be measured by a competition index (Uriarte et al., 2004; Zhu et al., 2017). Neighborhood is the local area where the objective tree is planted and grown in our study. We selected individuals of *Alniphyllum fortune*, *Machilus pauhoi* and *Castanopsis eyrei* with a diameter at breast height (DBH) greater than 5 cm in the plots as the object of analysis, and extended a spatial range of 6 m from each focal tree as the neighborhood (Yang et al., 2013; Duan et al., 2005). The competition index was calculated based on information including DBH, height (H), and tree coordinates of target trees, as well as those of the neighborhood trees. The function was as follows (Yang et al., 2013; Chen et al., 2016):

${Dist}_{ij}=\sqrt{\left( X_{i}-X_{j} \right)^{2}+\left( Y_{i}-Y_{j} \right)^{2}}$ (1)

${CI}_{ij}=\sum_{i=1}^{n} \left( \frac{D_{i}^{2}H_{i}}{{D_{j}^{2}H_{j}Dist}_{ij}} \right)$ (2)

where *Dist_ij_* represents the distance between *i* and *j*, *X_i_* and *Y_i_* are the *X* and *Y* coordinate values of neighboring *i* in the plot; *X_j_* and *Y_j_* are the *X* and *Y* coordinate values of target tree *j* in the plot, respectively; *CI_ij_* is the target tree species *j* faces competition intensity from neighboring trees; *n* is the number of individuals in neighboring species; *D_i_* is the DBH of adjacent individual *i*; *H_i_* is the height of adjacent individual *i*; *D_j_* is the DBH of target individual *j*; and *H_j_* is the height of target individual *j.*

We used independent sample t-tests to analyze differences in the received competitive intensity of the three species between BABF and BEBF. We found that bamboo expansion increases the competitive pressure on surviving broad-leaf trees (Table S1). Competitive intensity of *A. fortunei*, *M. pauhoi*, and *C. eyrei* in the BABF was 8.1 ± 4.2, 10.5 ± 1.8, and 12.3 ± 5.3, respectively, while in the BEBF it was 8.7 ± 4.2, 12.6 ± 6.6, and 35.5 ± 8.0, respectively.

Table S1. Competitive intensity of three surviving broadleaf tree species in BABF and BEBF.

| Stand | *Alniphyllum fortunei* | *Machilus pauhoi* | *Castanopsis eyrei* |
| --- | --- | --- | --- |
| BABF | 8.12 ± 4.22 | 10.52 ± 1.78 | **12.25 ± 5.29*** |
| BEBF | 8.70 ± 4.24 | 12.58 ± 6.55 | **35.53 ± 8.04*** |

References

Chen, Y. X., Wright, S. J., Muller-Landau, H. C., Hubbell, S. P., Wang, Y. F., and Yu, S. X. (2016). Positive effects of neighborhood complementarity on tree growth in a Neotropical Forest. *Ecology* 97, 776-785. doi: 10.1890/15-0625.1

Duan, R. Y., and Wang, X. A. (2005). Intraspecific and interspecific competition in larix chinensis. *Chin. J. Plant Ecol*. 29, 242-250.

Uriarte, M., Canham, C. D., Thompson, J., and Zimmerman, J. K. (2004). A neighborhood analysis of tree growth and survival in a hurricane-driven tropical forest. *Ecol. Monogr.* 74, 591-614. doi: 10.1890/03-4031

Yang, Q. P., Fu, F. L., Zang, L., Liang, Y. L., Tang, P. R., Liu, Z. G., et al. (2013). A study on the neighborhood interrerence index in *Toona ciliates* var. *pubescens* Community. *Acta Agric. Univ. Jiangxiensis* 35, 748-754. doi: 10.13836/j.jjau.2013132

Zhu, Y., Hogan, J. A., Cai, H. Y., Xun, Y. H., Jiang, F., and Jin, G. Z. (2017). Biotic and abiotic drivers of the tree growth and mortality trade-off in an old-growth temperate forest. *Forest EcoL. Mang.* 404, 354-360. doi: 10.1016/j.foreco.2017.09.004
